# VestAid: A Tablet-Based Technology for Objective Exercise Monitoring in Vestibular Rehabilitation

**DOI:** 10.3390/s21248388

**Published:** 2021-12-15

**Authors:** Pedram Hovareshti, Shamus Roeder, Lisa S. Holt, Pan Gao, Lemin Xiao, Chad Zalkin, Victoria Ou, Devendra Tolani, Brooke N. Klatt, Susan L. Whitney

**Affiliations:** 1 NxtHealth Team, Intelligent Automation, Rockville, MD 20855, USA; sroeder@i-a-i.com (S.R.); lholt@i-a-i.com (L.S.H.); pgao@i-a-i.com (P.G.); lxiao.fn@i-a-i.com (L.X.); czalkin@i-a-i.com (C.Z.); vou@i-a-i.com (V.O.); dtolani@i-a-i.com (D.T.); 2 School of Health and Rehabilitation Sciences, University of Pittsburgh, Pittsburgh, PA 15219, USA; bnk12@pitt.edu (B.N.K.); whitney@pitt.edu (S.L.W.)

**Keywords:** vestibular rehabilitation, VORx1 exercises, dizziness, exercise monitoring, telemedicine

## Abstract

(1) Background: Current vestibular rehabilitation therapy is an exercise-based approach aimed at promoting gaze stability, habituating symptoms, and improving balance and walking in patients with mild traumatic brain injury (mTBI). A major component of these exercises is the adaptation of the vestibulo-ocular reflex (VOR) and habituation training. Due to acute injury, the gain of the VOR is usually reduced, resulting in eye movement velocity that is less than head movement velocity. There is a higher chance for the success of the therapy program if the patient (a) understands the exercise procedure, (b) performs the exercises according to the prescribed regimen, (c) reports pre- and post-exercise symptoms and perceived difficulty, and (d) gets feedback on performance. (2) Methods: The development and laboratory evaluation of VestAid, an innovative, low-cost, tablet-based system that helps patients perform vestibulo-ocular reflex (VORx1) exercises correctly at home without therapist guidance, is presented. VestAid uses the tablet camera to automatically assess patient performance and compliance with exercise parameters. The system provides physical therapists (PTs) with near real-time, objective (head speed and gaze fixation compliance), and subjective (perceived difficulty and pre- and post- exercise symptoms) metrics through a web-based provider portal. The accuracy of the head-angle and eye-gaze compliance metrics was evaluated. The accuracy of estimated head angles calculated via VestAid’s low-complexity algorithms was compared to the state-of-the-art deep-learning method on a public dataset. The accuracy of VestAid’s metric evaluation during the VORx1 exercises was assessed in comparison to the output of an inertial measurement unit (IMU)-based system. (3) Results: There are low mean interpeak time errors (consistently below 0.1 s) across all speeds of the VORx1 exercise, as well as consistently matching numbers of identified peaks. The spatial comparison (after adjusting for the lag measured with the cross-correlation) between the VestAid and IMU-based systems also shows good matching, as shown by the low mean absolute head angle error, in which for all speeds, the mean is less than 10 degrees. (4) Conclusions: The accuracy of the system is sufficient to provide therapists with a good assessment of patient performance. While the VestAid system’s head pose evaluation model may not be perfectly accurate as a result of the occluded facial features when the head moves further towards an extreme in pitch and yaw, the head speed measurements and associated compliance measures are sufficiently accurate for monitoring patients’ VORx1 exercise compliance and general performance.

## 1. Introduction

An estimated 35.4% of adults in the United States have some kind of vestibular dysfunction requiring medical attention [1,2]. This dysfunction usually results in dizziness and vertigo, which can impact daily life and is a major risk factor for falls. Fall incidents are significantly greater in individuals with vestibular hypofunction than in healthy individuals of the same age and result in enormous direct and indirect medical costs [1]. While vestibular dysfunctions affect all populations, servicemembers and veterans are at higher risk of being subject to mild traumatic brain injury (mTBI) leading to elevated reports of dizziness [3]. Servicemembers exposed to blasts have frequent complaints of vertigo, gaze instability, motion intolerance, and other symptoms consistent with peripheral vestibular pathology [3].

Current vestibular rehabilitation is an exercise-based approach aimed at promoting gaze stability, habituating symptoms, and improving balance and walking [4]. A major component of these exercises is the adaptation of the vestibulo-ocular reflex (VOR), which is the primary eye motion mechanism to stabilize scenes on the human retina during fast head motions. As a result of acute injury, the gain of the VOR is usually reduced, resulting in eye movement velocity that is less than head movement velocity. Consequently, as people move their heads, the image on which they are stabilizing their vision slips across the retina and causes blurred vision and dizziness [4]. The most common exercises provided for persons with complaints of dizziness and concussion are gaze stabilization or VORx1 adaptation exercises. In these exercises, patients perform active eye and head movements in a lighted room while focusing on a target. These exercises recalibrate the VOR through the concept of retinal slip [5].

An integral part of vestibular rehabilitation is an individualized home exercise program that a clinician prescribes to address a patient’s particular functional limitations and impairments [1,6]. Vestibular rehabilitation is most effective when applied in a customized fashion [7]. Evidence shows that patients who are compliant with home exercise regimens—following them for extended time periods—demonstrate greater improvement than those who do not [8]. However, providing patient-compliant, home-program vestibular rehabilitation exercises is challenging due to patients’ difficulty in understanding and following the instructions, their lack of motivation, a lack of feedback to the patient, and other adherence issues. There is a higher chance for the success of the home exercise program if the patient (a) understands the exercise procedure, (b) follows the therapists’ instructions, (c) and provides pre- and post-exercise symptoms and perceived difficulty between visits that the clinician can use to give feedback on performance (knowledge of results).

To the best of our knowledge, only a few low-cost technological aids have been designed to ensure the patients’ correct performance of gaze stabilization exercises at home. Huang et al. developed an iPod-based system [9] to measure the consistency of head movement speed with the prescribed frequency as the patients perform VORx1 exercises. These exercises consist of patients moving their heads in the pitch, yaw or roll planes, while fixating their gaze on a target in front of them as they move their head at a prescribed speed. The objective is to keep the target in focus while moving the head at the prescribed speed. There are different parameters that will affect the outcome of the exercises, which an experienced physical therapist (PT) will change over the course of the therapy based on the individual’s needs. These include the type/size of the target, the distance from the target, the speed and frequency of head movement, the plane of head movement, the base of support, and posture of the subject. Another line of research by Schubert et al. has produced StableEyes, a portable device that allows the conduct of a VOR gain adaptation technique, in which the patient visually tracks a target, which moves with programmable speeds synchronously with their head [10].

In this paper, we present the VestAid, an innovative, low-cost home-exercise system that helps patients follow the clinicians’ instructions and uses the tablet camera to automatically assess compliance in the performance of the VORx1 exercises. The system is implemented as a tablet-based app (extendable to phones and monitor displays) for the patient and a web-based portal for the PT. The PT inputs the parameters of the exercises (suggested dosage, direction and speed of head movement, optotype shape and type, contrast with background). Video instructions on the tablet help patients recall how to perform the VORx1 exercises and a metronome guides head speed during the exercises. The system collects symptom data before and after each exercise, which is then combined with objective performance data to provide feedback to both the patient and the PT. All of the data are stored in the cloud to support remote monitoring by the rehab provider. With the tablet camera, face and eye detection is used to analyze the accuracy of the head and eye movement during the exercise. Convolutional neural network (CNN) models are used to detect the face from the image, and extract landmark points (eye and nose positions). Head pose is computed using the face image and the selected facial landmarks. A peak detection algorithm is used to determine head-speed compliance. The system provides the PT with near real-time objective (head speed, gaze compliance) and subjective (pre- and post- exercise symptoms, perceived difficulty) metrics of compliance through a web-based provider portal. In addition, the system provides individualized, gamified feedback and rewards to help keep the patient engaged, motivated, and compliant throughout the therapy. 

In this paper, we describe lab-testing of the feasibility of the VestAid system by: (1) Testing the accuracy of the VestAid head-angle estimation algorithm by comparing its performance against HopeNet [11], a state-of-the-art deep-learning method that requires too much computational load for use in the tablet-based app; (2) testing the accuracy of the VestAid head-turn frequency determination method by comparing its performance to an inertial measurement unit (IMU) affixed to the back of the head during the conduct of the exercises; and (3) evaluating the eye-gaze compliance algorithm using a publicly available dataset.

## 2. Materials and Methods

### 2.1. Description of the System

**Figure 1:**
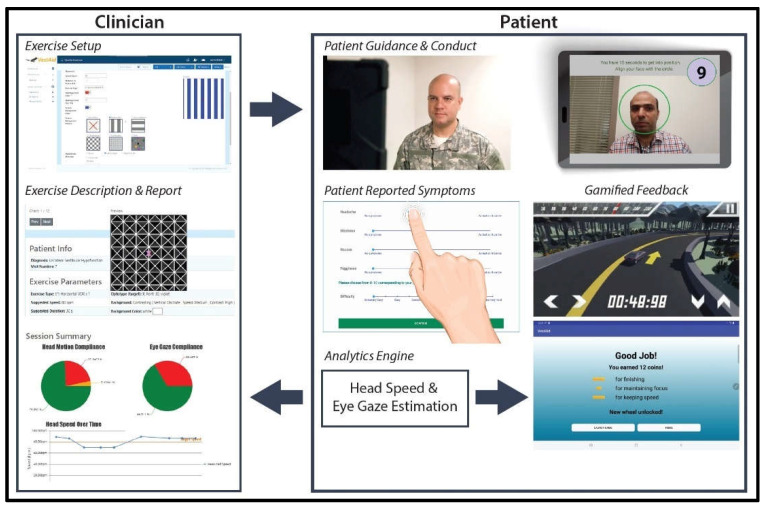
VestAid workflow.

The VestAid app provides adaptive individualized gaze-stabilization exercises based on therapist-provided directives. The PTs can design home program sessions and set the session parameters using the VestAid web portal, as shown in Figure 1. These parameters include the plane of head motion (yaw, pitch or roll), exercise dosing and duration, head speed, target size, background (type, color, stationary or moving, speed, and direction of motion), distance to target, and duration of the exercises. 

After the session parameters are set, the patients can use the VestAid app. The patient-facing tablet-based app provides exercise instructions through an instructional video and in-exercise guidance through metronome beeps tuned to the prescribed speed to ensure patients perform exercises properly at home. The tablet’s built-in camera is used to record the patient’s head movements and feeds the data to image-processing algorithms to track the patient’s eye-gaze, head movement range, and head velocity. The captured video is processed on the tablet to detect facial landmark points. VestAid estimates parameters of interest, such as 3D head angles (roll, pitch, and yaw) and eye movement in near real-time using novel machine learning algorithms. Based on these parameters, VestAid computes head and eye movement compliance metrics, such as the percentage of the patient that has performed the exercise with correct speed. The app also collects the patient’s pre- and post-exercise symptoms (dizziness, headache, nausea, and fogginess) and perceived difficulty of the exercise, all in formats from validated studies [12]. The computed head speed and eye-gaze compliance metrics as well as subjective symptoms are made available to the therapist via the VestAid web portal. To motivate the patient and improve exercise compliance, the therapist can enable a gamified reward system. The data are stored in the cloud to support remote monitoring by the PT. A summary of the VestAid app functionalities is provided in Table 1.

### 2.2. Description of Patient Interaction with the App

The App pages have been developed using the following guidelines:

Simple language and graphics: To minimize the reading of text material, wherever there is a text display, the language is kept simple (8th grade reading level), in order that it does not add to the patient’s cognitive load. In the body of the app, simple graphic displays and minimal animation are used to avoid the unwanted effect on patient symptoms. In addition, we developed videos to communicate instructions and demonstrate the exercises.

Contrast: The visual environment provided to the patients can affect their symptoms while doing the exercises. Therefore, we have avoided high-contrast and busy backgrounds to start and have selected muted and moderately contrasted background and font colors in the app to avoid unwanted patient symptoms. Busy backgrounds and high contrast settings are only available if the PT sets up an exercise, which requires these backgrounds.

Font size: Large fonts and a low density of text to display instructions/feedback is used to avoid cognitive fatigue.

**Figure 2:**
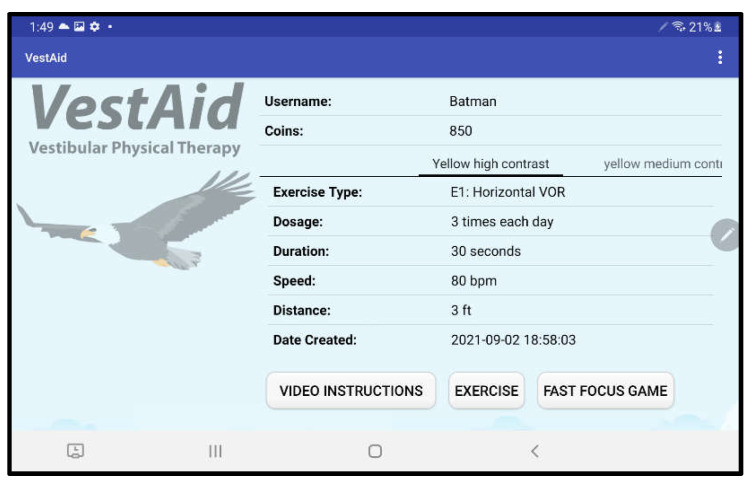
VestAid start page.

**Figure 3:**
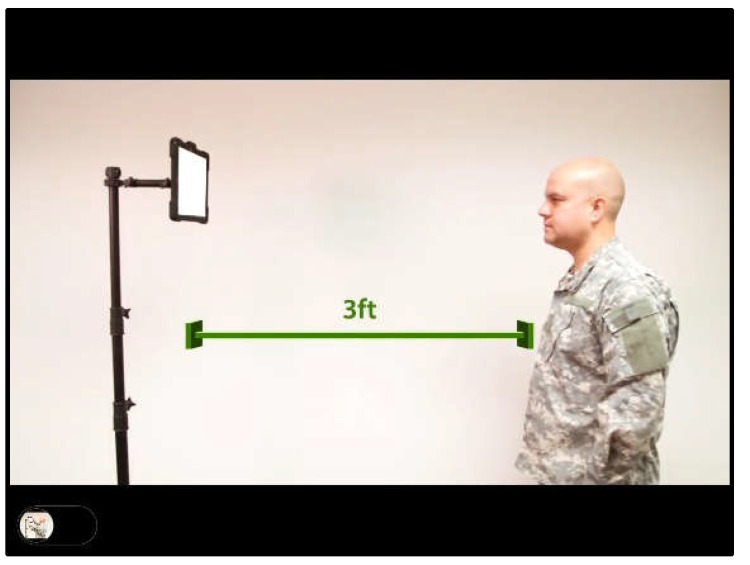
Instructional video page.

**Figure 4:**
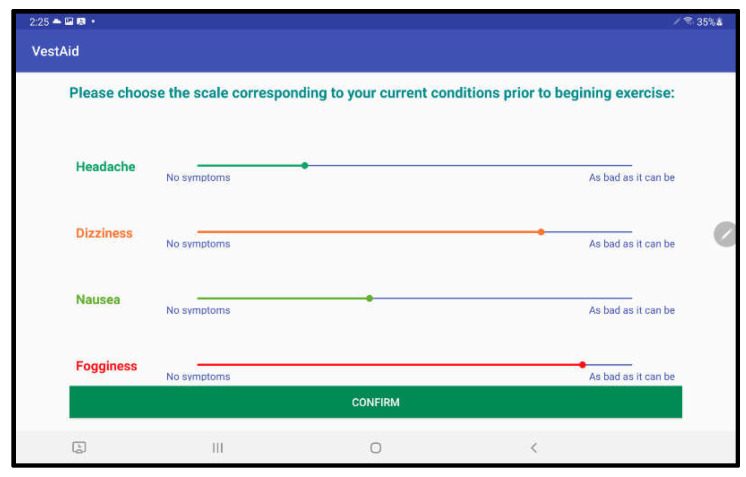
Pre-exercise symptom rating page.

**Figure 5:**
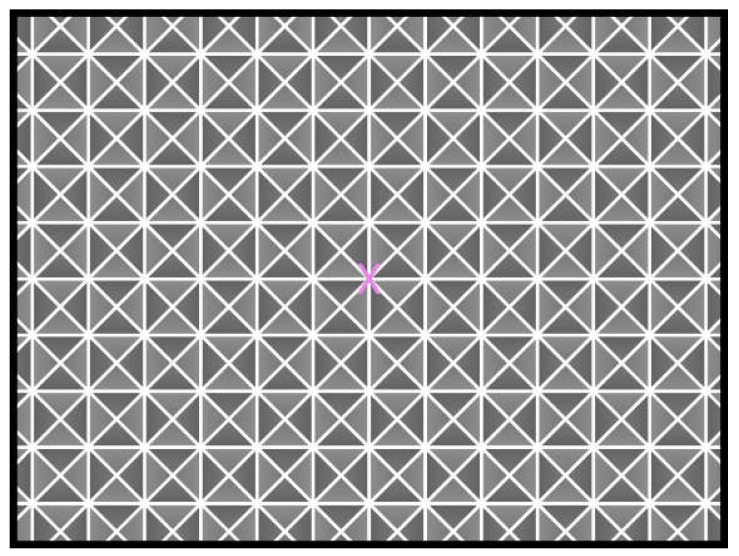
Exercise page (complex background).

**Figure 6:**
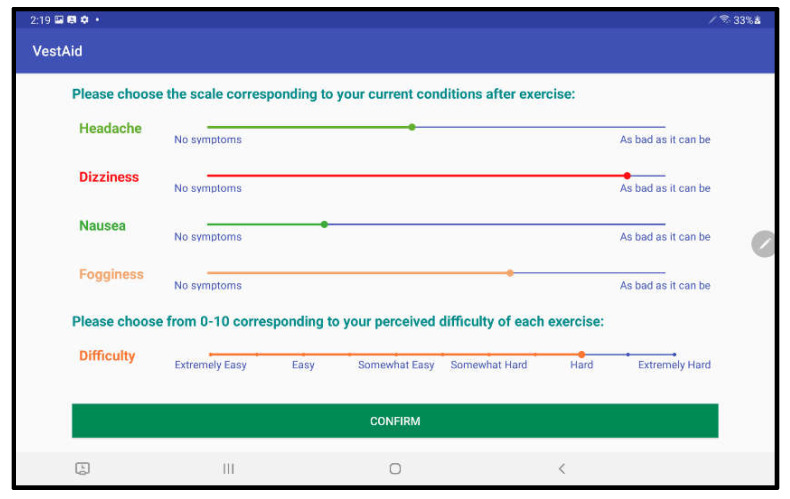
Post-exercise symptom rating page.

**Figure 7:**
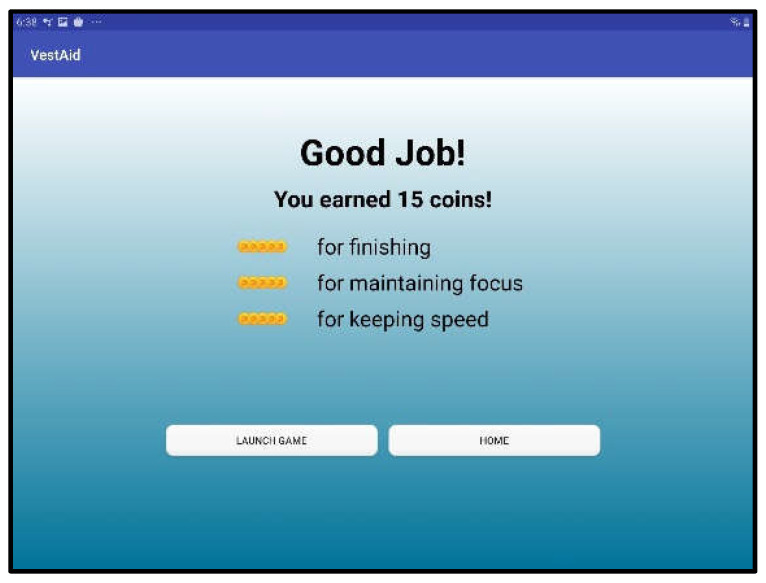
Feedback and reward page.

When patients log in to the app, they are taken to a start page that displays the summary of the prescribed exercises (Figure 2). From there, they can view a video that communicates instructions and demonstrates the exercises (Figure 3). Tapping on the exercise icon will take the patient to a page, in which they rate their pre-exercise dizziness, headache, nausea, and fogginess symptoms on a continuous scale of 0 (no symptom)–10 (as bad as it can get) based on the VOMS system of symptom rating [12] (Figure 4). Then, the patient is taken to the exercise screen with the background scene displayed using the patterns and contrasts set by the PT (Figure 5). After completing the exercise, the patient is led to a post-exercise screening page (Figure 6), in which they rate their post-exercise symptoms (similar to pre-exercise symptom rating), as well as the perceived difficulty, using a discrete scale of 0 (extremely easy)–10 (extremely hard) that has been validated by Robertson et al. ([13,14]). The patient is then presented with a feedback page with a performance and compliance reward summary (Figure 7). During the course of therapy, it is natural for patients to experience difficulty, in order for the rewards to not directly depend on an ideal performance. Instead, the patients are rewarded for various aspects of performance in order that every user will earn some reward so long as they attempt the exercise. Each patient will receive 5 virtual coins for completing the exercise, 0 to 5 coins based on their head speed compliance, and 0 to 5 coins based on their gaze compliance. These coins are incorporated into a reward-based system to encourage adherence to the therapy regimen.

### 2.3. Gamification

Gamification refers to the use of game design elements in non-game contexts [15]. The general idea is to use some elements, normally found to be motivating in games, for use in real-world situations to motivate certain behaviors, such as compliance to a repetitive rehabilitation exercise. Research results show that badges, leaderboards, and performance graphs positively affect user experience and task meaningfulness, while avatars, meaningful stories, and teammates affect the user’s relationship to social aspects of the situation (e.g., [16]). Gamification generally provides positive effects, but the effects depend on the context of the gamification and on the users using it [17]. Therefore, it is important to (1) choose gamification techniques that enhance the context, and (2) meet the needs of the users.

**Figure 8:**
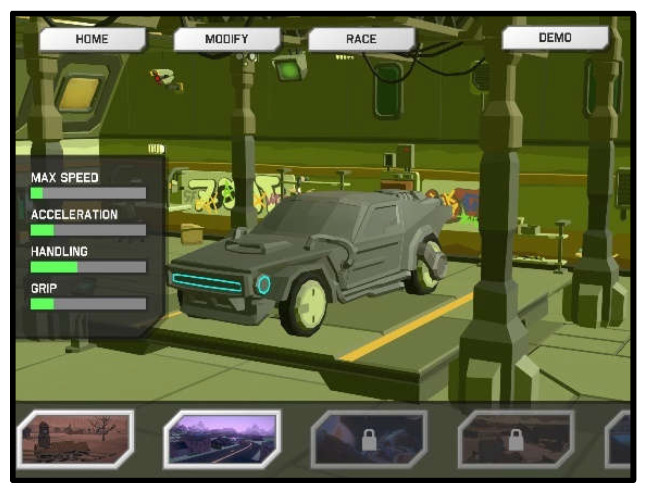
Garage scene.

**Figure 9:**
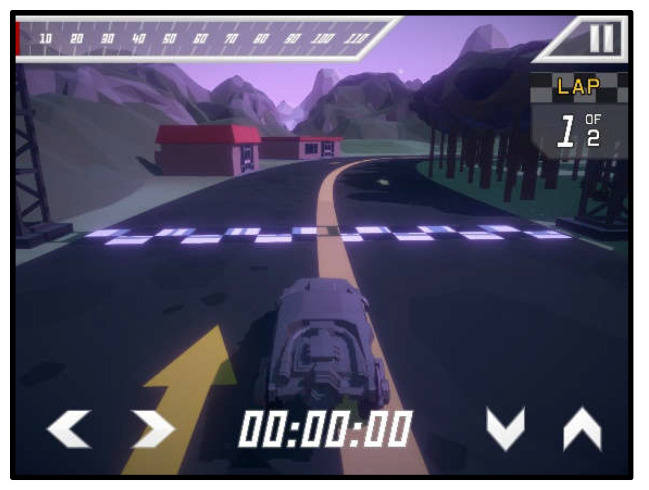
Race scene.

Since the VestAid technology is designed for the primary user population of post-concussion service members and athletes, a reward-based system with a car racing game component was designed. The racing game opens to a garage where the patient can spend the coins awarded based on their exercise performance to customize their racecar (Figure 8). The goal of each race is to finish in the shortest possible time (Figure 9). Patients gain access to the racing game by completing their required daily VORx1 exercises. For each day of completed exercises, patients will unlock a new functional upgrade or track in the game. Functional car upgrades make it easier to achieve the timing goals in each track. PTs can disable game functionality if they feel it will negatively affect a patient’s therapy. The goal of the game feature in the app is to provide a reward but it also serves a therapeutic purpose. When driving, the user is required to use smooth pursuits and saccades, and is exposed to visual backgrounds that can serve as a form of habituation training for the person living with dizziness.

### 2.4. Description of the Algorithms

To determine a patient’s head-motion and eye-gaze compliance in the VORx1 exercises, the captured video is analyzed on the tablet. Our approach includes the steps shown in the next subsections:

#### 2.4.1. Detection of the Patient’s Face in the Frames of the Captured Video

The first step in head and eye motion tracking is to detect the patient’s face in the video captured by the tablet’s camera. To this end, we have implemented a variant of the approach used by Liao et al. [18], which can effectively handle the unconstrained face detection situations, such as arbitrary pose variations and occlusions that can occur. To detect faces in the images, the approach uses an image feature called normalized pixel difference (NPD) to find the difference between the face and the background. This method uses a deep quadratic tree approach to learn the optimal sets of NDP features to partition the complex face manifolds. 

#### 2.4.2. Detection of Facial Landmarks and Estimation of Head Angles

**Figure 10:**
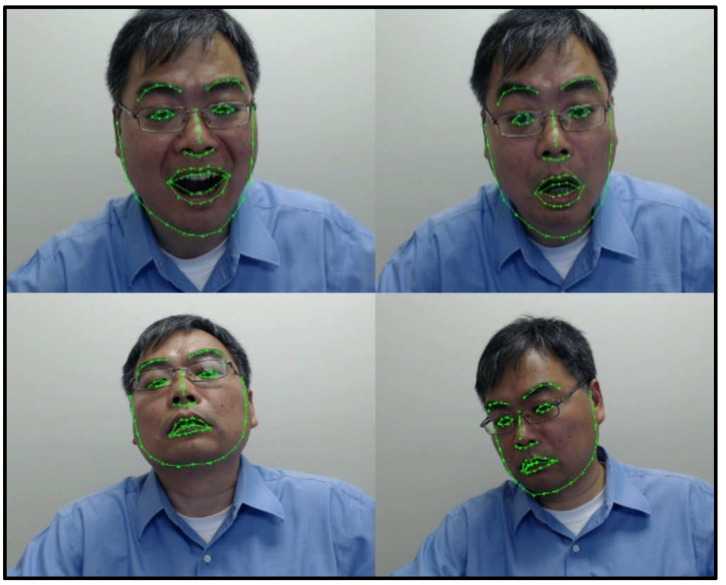
Facial landmark detection results based on SDM.

After the face is identified, the next step is to detect facial landmarks, such as the eyes (Figure 10). To this end, we have adopted and improved the method proposed by Ren et al. [19]. The approach aims to first perform encoding of the landmark-specific texture independently and then to do a joint regression. To improve the computational complexity, we have implemented a supervised descent method (SDM) based on the work by Xiong et al. [20] and Wu et al. [21]. 

Figure 10 shows a few examples, in which we detect different facial landmarks in the pictures, while the person in the frame changes head positions with respect to the camera, illustrating the robustness of the approach to changes in head position. 

#### 2.4.3. Determination of Head-Motion Compliance Based on the Estimated Head Angles

**Figure 11:**
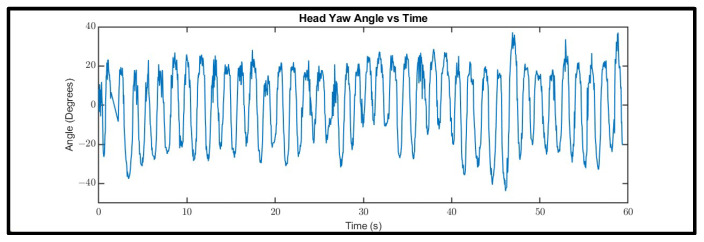
Head yaw motion signal for a 60-s horizontal VORx1 exercise.

The head motion in the VORx1 exercises will result in periodic head yaw (respectively pitch) signals when the patient does horizontal (respectively vertical) movements (Figure 11).

The head angles oscillate between two extremes, represented by peaks and valleys in the curve. If the head movements are performed uniformly with exactly the same amplitude and frequency, the head angle waveform would be a perfectly periodic curve with the period equal to the period of head movement and the amplitude equal to the amplitude of the head movement (maximum head angle displacement from the neutral position, in which one’s head has zero yaw angle). In reality, the motion is not perfectly periodic as the subject’s slightest change in head movement speed will cause changes in the peak-to-peak (valley-to-valley) time, which represents the time of a left-to-right-to-left (or right-to-left-to right) head movement.

Since the sampling is not uniform (i.e., the time difference between two consecutive frames is not constant and can vary by a few milliseconds), we did not apply Fourier-based methods to determine head speed compliance. Instead, we used a computationally efficient peak detection algorithm that smooths the signal before identifying the local maxima [22]. Following this, the generated list of peaks is further filtered by only retaining peaks with a prominence greater than their height, thereby only leaving the greatest local maxima within each region bounded by a zero-crossing on each side. Finally, all peaks with a height under an arbitrary threshold (4 degrees) are dropped to ensure that a small perturbation is not treated as a full head turn. Using the above method, we extract the peaks and valleys of the filtered signal, and determine the actual head movement speed using the time difference between peaks and valleys. To decide whether the patient has complied to the prescribed speed, we define a compliance range (Δ) to be within 15 bpm of the prescribed speed. For example, if the prescribed exercise requires a head movement speed of 120 metronome bpm, there is a window of ±15 bpm around 120 bpm that would constitute an acceptable exercise speed. Any portion of the exercises that fall outside this range is marked slow or fast. 

#### 2.4.4. Determination of Eye-Gaze Compliance Based on Classification of Eye-Gaze Detection

**Figure 12:**
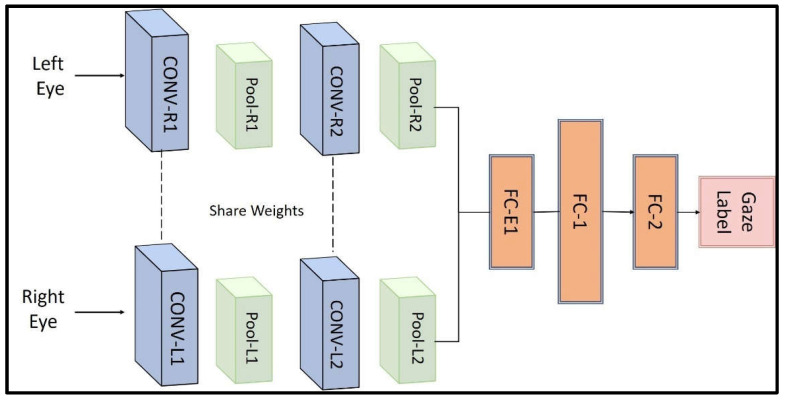
Architecture of CNN-based eye-gaze estimation algorithm.

To determine eye-gaze compliance, we used a light-weight deep learning-based method that is able to run on a regular tablet or smartphone without the need for a graphics processing unit (GPU). We developed a convolutional neural network (CNN)-based module for classifying the eye-gaze direction as “on-target” or “off-target” based on the work of [23]. Figure 12 shows the original architecture of the neural networks that employs convolutional layers followed by fully-connected (FC) layers. 

**Figure 13:**
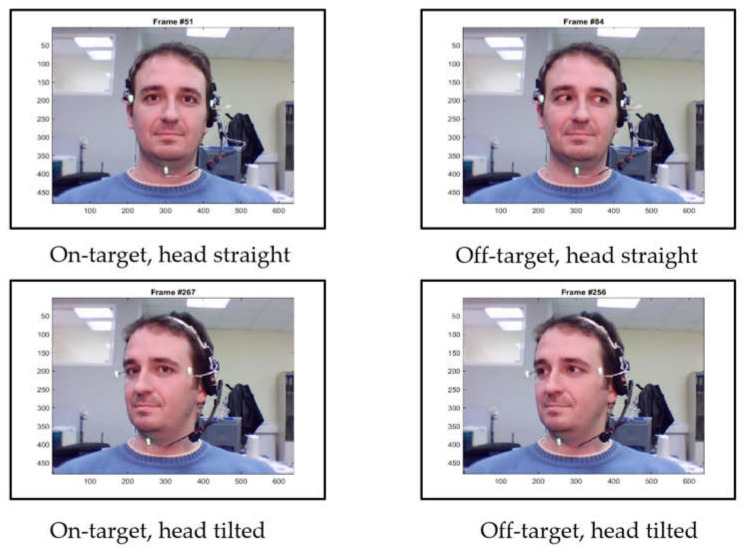
Example gaze labeling in videos of the public dataset from [24].

To train the network, a public dataset [24] of videos of 10 persons conducting head and eye motions similar to VORx1 exercises was used. Frames from the videos were extracted and labeled as “on-target” and “off-target” based on whether the gaze was directed towards the target regardless of head orientation (Figure 13). Left and right eye images were used as the network input and the output is the binary classification result. 

We tuned the network using various network structures with different numbers of convolutional layers or fully connected layers and compared the measures associated with performance metrics to finalize the network structure. Table 2 shows the performance comparison of five different network structures. The network with two CNN layers for each eye and three fully connected layers reached the best accuracy, precision, and F1 score ([25]), while the network with two CNN layers and two fully connected layers has the best recall. Taking all metrics into consideration, we chose the network with two CNN layers and three fully connected layers and implemented it in the VestAid software.

### 2.5. Evaluating the Accuracy of Head-Angle Estimation

**Figure 14:**
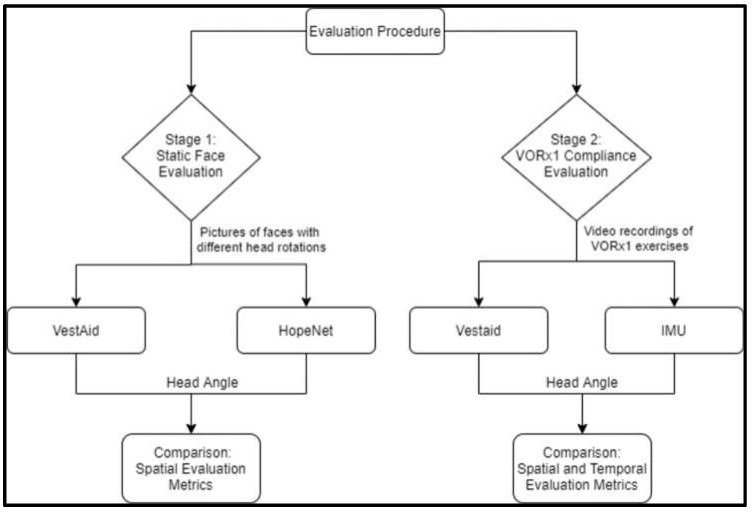
Overview of two-stage evaluation procedure.

We tested the accuracy of the VestAid head-angle estimation algorithm in two stages, as shown in the next subsection. Figure 14 outlines the overall procedure.

#### 2.5.1. Evaluation of Head-Angle Estimation on Static Faces from a Public Dataset 

The first stage was to compare the accuracy of estimated head angles against a state-of-the-art deep-learning method on a public dataset. Once we established that the algorithms perform well on static face figures from the public dataset, we conducted the second evaluation, i.e., the evaluation of the head-speed compliance metric resulting from the implementation of the algorithms in the app.

For the head angle evaluation, we compared the performance of VestAid against that of HopeNet [11], a state-of-the-art deep-learning that requires too much computational load for use in the tablet-based app. We ran both algorithms on a subset of the Biwi Kinect Head Pose Database, which contains head rotations in the range of ±75° (yaw), ±60° (pitch), and ±50° (roll) [26]. 

To compare the accuracy of the VestAid algorithm to the HopeNet, we examined the average absolute error of the individual extrinsic Euler angles, the mean squared error of all three extrinsic Euler angles, and the geodesic of the quaternion representations of the true and estimated final orientations of the head with the given Euler angles for each model. 

Details on the comparison between the two systems are included in the Supplementary Materials, Section S1 [26].

#### 2.5.2. Evaluation of Head Angles and Speed Compliance in Action

To evaluate the accuracy of VestAid’s metrics during the VORx1 exercises, the VestAid algorithm’s output was compared to the output of an inertial measurement unit (IMU)-based system using a ruggedized IMU device developed by IAI (Rockville, MD, USA). The IMU was affixed to the back of the head of the first author performing six variations of VORx1 exercises (Table 3) on the VestAid app.

**Figure 15:**
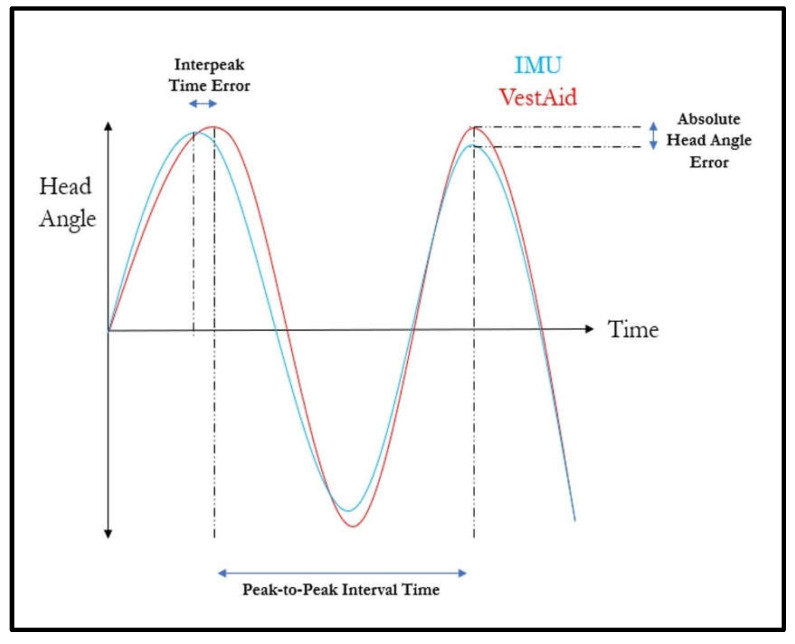
Temporal and spatial error metrics used to compare the VestAid and IMU-based system.

For each trial, the VestAid system was set to run for 30 s at 80, 120 or 160 bpm. The subject followed the system’s metronome as closely and as smoothly as possible and completed at least a 30-degree head sweep (+/− 15 degrees) for each head turn. Data recording with the IMU began when the head alignment countdown reached 5 s remaining and stopped once the exercise was completed. Five trials were completed at each target bpm for each direction (horizontal/vertical) for a total of 30 trials. The IMU and VestAid data were manipulated to the output head angle at timepoints of interest (details outlined in Supplementary Materials, Section S2 [27,28].). To quantify the accuracy of the head angle from the VestAid signal, the timepoints and their corresponding head angles from the VestAid signal were then compared to their matching timepoints on the IMU-derived signal to calculate the absolute error in head angle (Figure 15).

To examine the frequency metrics output from the VestAid signal, the difference in the timings between each peak (interpeak time error) for both the VestAid and IMU-derived signal was calculated (Figure 15). For the instances where one or two individual peaks may have been missed by the VestAid or IMU-derived signal, the corresponding peak in the other signal was dropped from the analysis of interpeak times. The total number of identified peaks, the measured head turn frequency, and the ‘percent correctness’ were also compared. While many different types of metrics were calculated and compared against the IMU-based system, the main goal of this validation study was to ensure that the VestAid system can correctly determine whether the patient is performing the VORx1 exercises correctly.

## 3. Results/Discussion

### 3.1. Evaluation of Head-Angle Estimation on Static Faces from a Public Dataset

On the Biwi dataset described in Section 2, the VestAid head-angle estimation performed comparably to HopeNet with less than 10 degrees average geodesic error, less than 8 degrees average absolute error in the pitch plane, and less than 6 degrees average absolute error in the yaw plane, as shown in Table 4. Therefore, the VestAid system shows a good capacity to accuracy estimate head angles on static faces.

### 3.2. Evaluation of Head Speed Compliance

Table 5 summarizes the spatial and temporal error calculations of the VestAid system with reference to the IMU-based derivation. 

As shown in Table 5, the peak detection of the VestAid system appears to align reasonably well with the peaks derived from the IMU-based system. There are low mean interpeak time errors (consistently below 0.1 s) across all speeds of the VORx1 exercise, as well as consistently matching numbers of identified peaks. The spatial comparison (after adjusting for the lag measured with the cross-correlation) between the VestAid and IMU-based systems also show good matching, as shown by the low mean absolute head angle error, in which for all speeds, the mean is less than 10 degrees. Incidences of higher error in head angle are likely due to noisy head-angle calculations from the face-detection algorithm.

**Figure 16:**
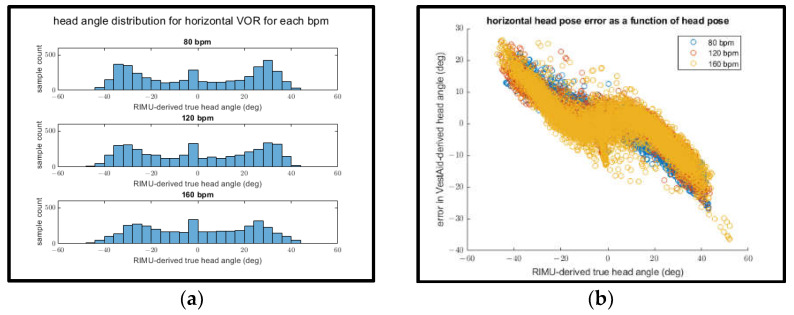
(**a**) IMU-derived head angle distribution for all trials of horizontal VORx1 exercises at different bpms; (**b**) error in the horizontal head pose angle vs. IMU-derived head angle.

**Figure 17:**
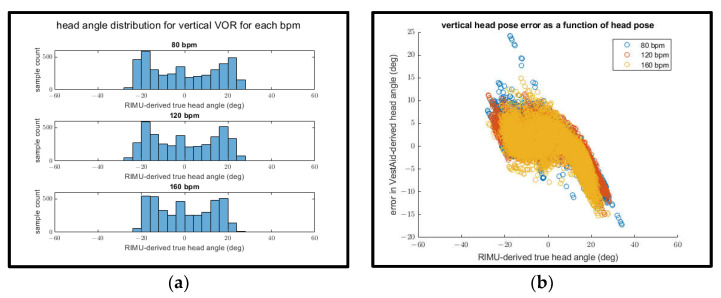
(**a**) IMU-derived head angle distribution for all trials of vertical VORx1 exercises at different bpms; (**b**) error in the vertical head pose angle vs. IMU-derived head angle.

Interestingly, when examining the mean absolute head-angle error and the mean RMSE for both the horizontal and vertical directions, the error appears to decrease as the goal bpm increases. This can be explained by plotting both the distribution of IMU-derived head angles for each goal bpm (Figure 16a and Figure 17a) and the relationship between the IMU-derived head angles and the error of the VestAid-derived head angles (Figure 16b and Figure 17b).

In both the vertical and horizontal directions (as shown in Figure 16a and Figure 17a), the range of motion of the subject appears to decrease as the goal bpm increases. As the IMU-derived head angle increases in magnitude (as shown in Figure 16b and Figure 17b), the magnitude of the error also increases. This suggests that the decrease in apparent head-angle error as the goal bpm increases is attributable to the subject’s decreased range of motion at higher bpm rather than the higher bpm itself leading to improved performance. 

The mean absolute head turn frequency error was the highest for the horizontal VORx1 exercise performed at 160 bpm, with a value of 10.77 bpm. As previously stated, the bounds for what is considered as ‘correct’ are set at +/− 15 bpm. Therefore, this is considered acceptable when providing feedback to the user as to whether they are performing the exercise properly. One critical aspect is that the error in these head turn frequency measurements is a function of the error in the measurement of the constituent peak-to-peak time interval (s) and the goal bpm, as shown in Equation (1). The derivation for Equation (1) is demonstrated in Supplementary Materials, Section S3.

(1) $$err_{bpm}=goal\left(-\frac{err_{interval}}{\frac{120}{goal}+err_{interval}}\right)$$

As the goal bpm increases, the same error in the measurement of the peak-to-peak interval produces a higher error in the final bpm measurement.

When evaluating the performance of a subject for a specific trial, the VestAid and IMU system give comparable metrics on the percentage of the exercise conducted correctly, as shown by the similar mean correct percent values in Table 5. For trials condutced at 80 bpm, the difference between the mean correct percent values is as low as 0.04 and 0.06 for the horizontal and vertical tests, respectively. For higher bpms, the difference between the two systems is greater, due to the aforementioned mathematical relationship between the peak-to-peak interval measurement error and the frequency measurement (Equation (1)), but still shows as being close in performance, with differences of less than 12.3% across all head speeds (bpm). Overall, these results demonstrate VestAid’s capability to accurately evaluate whether a patient is currently performing a VORx1 exercise at the assigned speed.

There were a number of limitations and challenges across these evaluation exercises. First and foremost, the ground truth on which this analysis is based was derived via the IMU measurement rather than the gold standard of a motion capture system. This ground truth also incorporated the mean measured head angle from the VestAid system in order to compensate for the lack of a DC-component to the IMU-derived head angle. Finally, this ground truth was temporally aligned with the VestAid signal through the use of cross-correlation, which has been previously employed to align camera-based position and IMU signals within VR systems [27,28]. While visual inspection was done on each trial to ensure that the two signals appeared to be correctly aligned using this method, it is important to note that this is not an ideal method of alignment for validation purposes when compared to a perfect external synchronization without lag and was employed to correct for a consistent time the lag encountered between the signals when external synchronization was used. 

## 4. Summary

In this paper, we demonstrated the capabilities and an initial evaluation of VestAid, an innovative, low-cost, tablet-based system that helps patients perform vestibulo-ocular reflex exercises (VORx1) correctly at home without therapist guidance. VestAid uses the tablet camera to automatically assess patient performance and compliance with exercise parameters. The system provides PTs with near real-time objective (head speed and gaze fixation compliance) and subjective (perceived difficulty and pre- and post- exercise symptoms) metrics through a web-based provider portal. In addition, the system uses gamification to help keep the patient engaged throughout the therapy.

Initial results suggest that the accuracy of the system is sufficient to provide PTs with an accurate and quantitative assessment of patient performance. Systematic data collection and human studies to further validate the technology are underway. While the VestAid system’s head-pose evaluation model may not be perfectly accurate as the head moves further towards an extreme in any direction as a result of the occluded facial features, the final head speed measurements and associated compliance measures are accurate for the purposes of monitoring patient exercise compliance and general performance.

In the future, it would be worthwhile to further evaluate VestAid’s capabilities against a gold-standard model for head pose detection. Furthermore, an investigation into ways to improve head-angle estimation at more extreme head angles could help improve the spatial accuracy of the system.

## 5. Patents

A patent application with USPTO was filed on 8 September 2020.

## Figures and Tables

**Table 1:** Summary of VestAid software functionalities.

Functionality	Implementation
Exercise setup	The therapist can easily set individualized exercise parameters for VORx1 exercises in the VestAid web portal: Exercise duration and dosing (no. of times/day); distance from the screen; screen background; size, color, and attributes of optotypes; and frequency of head movement.
Exercise guidance	The app includes instructional videos to help patients understand how to perform the exercises. The app guides the patients during the exercises by providing audio metronome beeps with the prescribed frequency. Audio beeps are played in the app similar to a metronome with an adjustable beat per minute (bpm) rate. The PT sets the bpm rate according to the required frequency of head movement with a default value of 1–2 Hz as supported by research [1,12].
Objective and subjective data collection	VestAid computes objective measures of the patients’ head motion and eye-gaze compliance (from video captured by the tablet camera during the exercise). VestAid collects pre- and post-exercise subjective symptom ratings (headache, dizziness, nausea, and fogginess) based on vestibular/ocular-motor screening (VOMS) for concussion [12]. At the end of each exercise, VestAid collects patients’ ratings of the perceived difficulty.
Compliance determination	Machine learning algorithms determine patients’ facial features and head angles. Based on these features, compliance of head motion (percentage of time conducted with prescribed speed vs. fast or slow; change of the head speed as a function of time) and eye-gaze (percentage of time focusing on the optotype target) are determined.
Patient feedback	Feedback on exercise compliance is provided to patients using an encouraging game-based rewards system. If enabled by the PT, patients can spend their exercise rewards in a computerized racing game.
PT reports	Easy-to-understand, time-stamped reports with graphical summaries are generated for the therapist. The PT can access reports through the web portal.

**Table 2:** Comparison of different neural network structures.

Network	Confusion Matrix				Accuracy	Precision	Recall	F1 Score
Two layers of CNN for each eye, three fully connected layers			Prediction		0.9428	0.9296	0.8989	0.9140
			Off-target	On-target				
	Ground Truth	Off-target	2002	72				
		On-target	107	951				
One layer of CNN for each eye, three fully connected layers			Prediction		0.9176	0.8984	0.8526	0.8749
			Off-target	On-target				
	Ground Truth	Off-target	1972	102				
		On-target	156	902				
Three layers of CNN for each eye, three fully connected layers			Prediction		0.9256	0.8852	0.8960	0.8906
			Off-target	On-target				
	Ground Truth	Off-target	1951	123				
		On-target	110	948				
Two layers of CNN for each eye, two fully connected layers			Prediction		0.9412	0.9092	0.9178	0.9135
			Off-target	On-target				
	Ground Truth	Off-target	1977	97				
		On-target	87	971				
Two layers of CNN for each eye, four fully connected layers			Prediction		0.9345	0.9074	0.8979	0.9026
			Off-target	On-target				
	Ground Truth	Off-target	1977	97				
		On-target	108	950				

**Table 3:** Exercise attributes used in the evaluation.

Task	Direction	Speed (bpm)
1	Horizontal	80
2	Horizontal	120
3	Horizontal	160
4	Vertical	80
5	Vertical	120
6	Vertical	160

**Table 4:** VestAid and HopeNet head-angle estimation accuracy on a subset of Biwi dataset.

Model	Avg abs Pitch Error (deg.)	Avg abs Yaw Error (deg.)	Avg abs Roll Error (deg.)	Avg Geodesic (deg.)
HopeNet	4.89	8.47	4.00	10.27
VestAid	7.61	5.98	4.91	9.65

**Table 5:** Overview of temporal and spatial error metrics of the VestAid system.

Direction	Horizontal			Vertical		
Speed (bpm)	80	120	160	80	120	160
No. of trials in category	5	5	5	5	5	5
Mean abs head angle error (deg.)	9.12	7.29	6.55	4.09	3.66	3.36
Mean head angle RMSE (deg.)	10.46	8.92	8.05	5.08	4.49	4.15
Mean no. of ID’d IMU peaks	17.00	26.00	32.40	17.00	25.60	33.40
Mean no. of ID’d VestAid peaks	17.00	26.00	31.80	16.80	25.20	33.20
Mean matched interpeak time error (s)	0.06	0.08	0.05	0.09	0.07	0.04
Mean matched interpeak time RMSE (s)	0.07	0.14	0.07	0.15	0.12	0.07
Mean abs head turn frequency error (bpm)	3.08	9.02	10.77	4.42	8.17	8.51
Mean head turn frequency RMSE (bpm)	3.79	12.44	13.62	6.21	11.37	13.00
Mean correct percent IMU (%)	99.52	98.67	97.47	98.76	98.76	91.13
Mean correct percent VestAid (%)	99.56	86.98	85.20	98.82	90.67	80.67
Mean correct percent difference (%)	0.04	−11.69	−12.27	0.06	−8.08	−10.46

## Data Availability

Publicly available datasets were used for training and initial testing of the software in this study. These data are referenced in the text. The rest of the data acquired for lab testing are available on request from the corresponding author.

## Supplementary Materials

The following are available online at https://www.mdpi.com/article/10.3390/s21248388/s1, Section S1: Details on the evaluation of head-angle estimation on static faces in a public dataset; Section S2: Details on the evaluation of head angles and speed compliance in action; Section S3: Derivation of the bpm error function (Equation (1)).
