# Supplementary material for: VestAid: A Tablet-Based Technology for Objective Exercise Monitoring in Vestibular Rehabilitation
Source: Sensors (Basel). 2021 Dec 15;21(24):8388. doi: 10.3390/s21248388 (PMC8706065; doi:10.3390/s21248388)
Supplement: Supplementary file 1 [file sensors-21-08388-s001.zip › sensors-1502079-supplementary.pdf]

sensors

MDPI

Article

VestAid: A Tablet-Based Technology for Objective Exercise Monitoring in Vestibular Rehabilitation

Pedram Hovareshti [1,*], Shamus Roeder [1], Lisa S. Holt [1], Pan Gao [1], Lemin Xiao [1], Chad Zalkin [1], Victoria Ou [1], Devendra Tolani [1], Brooke N. Klatt [2] and Susan L. Whitney [2]

1 NxtHealth team, Intelligent Automation, Rockville, MD 20855, USA; sroeder@i-a-i.com (S.R.); lholt@i-a-i.com (L.S.H.); pgao@i-a-i.com (P.G.); lxiao.fn@i-a-i.com (L.X.); czalkin@i-a-i.com (C.Z.); vou@i-a-i.com (V.O.); dtolani@i-a-i.com (D.T.)
2 School of Health and Rehabilitation Sciences, University of Pittsburgh, Bridgeside Point 1, 100 Technology Drive, Suite 210, Pittsburgh, PA 15219, USA; bnk12@pitt.edu (B.N.K.); whitney@pitt.edu (S.L.W.)
* Correspondence: phovareshti@i-a-i.com

Citation: Hovareshti, P.; Roeder, S.; Holt, L.S.; Gao, P.; Xiao, L.; Zalkin, C.; Ou, V.; Tolani, D.; Klatt, B.N.; Whitney, S.L. VestAid: A Tablet-Based Technology for Objective Exercise Monitoring in Vestibular Rehabilitation. *Sensors* **2021**, *21*, 8388. https://doi.org/10.3390/s21248388

Academic Editor: Marco Iosa

Received: 24 November 2021
Accepted: 11 December 2021
Published: 15 December 2021

Publisher's Note: MDPI stays neutral with regard to jurisdictional claims in published maps and institutional affiliations.

Copyright: © 2021 by the authors. Licensee MDPI, Basel, Switzerland. This article is an open access article distributed under the terms and conditions of the Creative Commons Attribution (CC BY) license (https://creativecommons.org/licenses/by/4.0/).

Supplementary Materials: The following are available online at www.mdpi.com/article/10.3390/s21248388/s1, Section S1: Details on the evaluation of head-angle estimation on static faces in a public dataset; Section S2: Details on the evaluation of head angles and speed compliance in action; Section S3: Derivation of the bpm error function (Equation (1)).

S1: Details on the Evaluation of Head-Angle Estimation on Static Faces in a Public Dataset

For the head angle comparison, we ran both algorithms (VestAid and HopeNet) on a subset of the Biwi Kinect Head Pose Database, which contains head rotations in the range of ±75° (yaw), ±60° (pitch), and ±50° (roll) [26]. Because of the extended range of head rotations, many of the dataset pictures were not well-suited to simulate faces from VORx1 exercises; therefore, we manually selected 1066 frames from this dataset.

Evaluation metrics to compare the two systems include the average absolute error of the individual extrinsic Euler angles, the mean squared error of all three extrinsic Euler angles, and the geodesic of the quaternion representations We used multiple error metrics for future comparison with other studies into head-pose estimation model accuracy and, in the case of the geodesic, to ensure that one of our error metrics would be unaffected by a conversion between intrinsic and extrinsic Euler angles. In the case of a model returning a null value for a frame, meaning that no face was detected, these frames were ignored.

S2: Details on the Evaluation of Head Angles and Speed Compliance in Action

The IMU device directly outputs the angular velocity of the subject's head at 225 Hz. The angular velocity was integrated and high-pass filtered at 0.1 Hz to derive the head angle. For the purposes of this analysis, the IMU-derived head angle signal and metrics are treated as the ground truth, albeit without a DC-offset.

In order to compare the IMU-derived head angle with the VestAid system output, the two timeseries need to be synchronized. Because the VestAid system outputs head angles at a much lower rate than the IMU device (25 Hz), the VestAid signal was upsampled at 225 Hz using spline interpolation to match the frequency of the IMU output. The temporal displacement between the two signals was calculated by finding the lag at the maximum cross-correlation between the resampled VestAid output and the IMU-derived head angle. (This method has been previously employed in general temporal signal alignment [27] and to align camera-based position and IMU signals within VR systems [28]) The two datasets could then be temporally aligned using the calculated temporal displacement. To align the two datasets in the spatial plane, the mean value of the VestAid output was added to the IMU-derived head angle signal to restore the aforementioned missing DC-offset.

To quantify the accuracy of the head angle from the VestAid signal, the original timepoints and their corresponding head angles from the VestAid signal (25 Hz) were then compared to their matching timepoints on the IMU-derived signal to calculate the absolute error in head angle. The additional up-sampled VestAid values were not used during the comparison to ensure that only the values directly provided by the VestAid system were being evaluated.

S3: Derivation of the Bpm Error Function (Equation (1))

The core metric used by physical therapists when evaluating patients' performance in VOR exercises and their prescription thereof is the head turn frequency as measured in beats per minute (bpm). The goal bpm is applied through a metronome during the VOR exercise, for which the patient will attempt to have their head reach either extreme (left-right or up-down) on each beat. Because this clinical bpm measurement describes the frequency with which their head movement completes one half-cycle (from one extreme to the other), the true signal frequency describing a completed cycle (to go from one extreme to the other and back again), is half that. For example, if the goal bpm is set to 120 bpm, over the course of a 1-minute VOR exercise a patient performing the exercise perfectly will move their head to the left 60 times and to the right 60 times, alternating each time. From the perspective of one examining the signal of the patient's head angle with respect to time, this head movement would be described as occurring at a 1 Hz frequency. For clarity, all uses of "bpm" within this derivation describe the "clinician's bpm" measurement as ${}^{1}/_{120}\ s^{-1}$ while "Hz" refers to a standard Hz measurement of $1s^{-1}$. This in turn gives us the conversion factor shown in Equation S1 below.

$$\frac{120bpm}{1Hz} = 1 \tag{1}$$

In addition, our emphasis in this characterization of error is on how our system would measure a patient's error in performing the VOR exercise to a prescribed bpm, not our system's error in making this measurement. From the prescribed goal bpm (denoted $goal_{bpm}$), we derive an expected (or goal) peak-to-peak interval time (denoted $goal_{interval}$ and measured in seconds) via our previously described unit conversion, shown in Equation S2 below. A description of this peak-to-peak interval time can be found in the main body of this paper in Section 2.5.2. Evaluation of head angles and speed compliance in action.

$$goal_{interval} = \frac{1}{goal_{bpm}} * \left(\frac{120\ bpm}{1Hz}\right) \tag{2}$$

The measured peak-to-peak interval time from the patient (denoted $patient_{interval}$) provided by our system can be used to generate our measured bpm (denoted $patient_{bpm}$) with the same unit conversion.

$$patient_{bpm} = \frac{1}{patient_{interval}} * \left(\frac{120\ bpm}{1Hz}\right) \tag{3}$$

Equations S4 and S5 below formalize our error definitions within this context.

$$err_{bpm} = patient_{bpm} - goal_{bpm} \tag{4}$$

$$err_{interval} = patient_{interval} - goal_{interval} \tag{5}$$

From here, we can derive our final error in bpm measurement (denoted err_{bpm}) as a function of the error in the peak-to-peak interval (denoted $err_{interval}$) and the set goal bpm, beginning with Equation S4 above.

$$err_{bpm} = patient_{bpm} - goal_{bpm}$$

$$err_{bpm} = \left(\frac{1}{patient_{interval}} * \left(\frac{120\ bpm}{1Hz}\right)\right) - goal_{bpm}$$

$$err_{bpm} = \left(\left(\frac{1}{goal_{interval} + err_{interval}}\right) * \left(\frac{120\ bpm}{1Hz}\right)\right) - goal_{bpm}$$

$$err_{bpm} = \left(\left(\frac{1}{\frac{1}{goal_{bpm}} * \left(\frac{120\ bpm}{1Hz}\right) + err_{interval}}\right) * \left(\frac{120\ bpm}{1Hz}\right)\right) - goal_{bpm}$$

We apply and condense the conversion factor, $\left(\frac{120\ bpm}{1Hz}\right)$, to 120 for readability before rearranging our terms.

$$err_{bpm} = \left(\frac{120}{\frac{120}{goal_{bpm}} + err_{interval}}\right) - goal_{bpm}$$

$$err_{bpm} = \left(\frac{120}{120/goal_{bpm}} - \frac{err_{interval} * 120}{\left(\frac{120}{goal_{bpm}}\right)^2 + \frac{120}{goal_{bpm}} * err_{interval}}\right) - goal_{bpm}$$

$$err_{bpm} = \left(goal_{bpm} - \frac{err_{interval}}{\frac{120}{goal_{bpm}{}^2} + \frac{1}{goal_{bpm}} * err_{interval}}\right) - goal_{bpm}$$

$$err_{bpm} = \left(goal_{bpm} - \frac{goal_{bpm} * err_{interval}}{\frac{120}{goal_{bpm}} + err_{interval}}\right) - goal_{bpm}$$

$$err_{bpm} = -\frac{goal_{bpm} * err_{interval}}{\frac{120}{goal_{bpm}} + err_{interval}}$$

This equation can now be rearranged and the $goal_{bpm}$ variable can be renamed to $goal$ for readability.

$$err_{bpm} = goal\left(-\frac{err_{interval}}{\frac{120}{goal} + err_{interval}}\right)$$
